# Are New Technologies suitable for Real-World Management of Dementia?

**DOI:** 10.1192/j.eurpsy.2025.161

**Published:** 2025-08-26

**Authors:** R. David

**Affiliations:** Memory Clinic – Sport-related concussion - Old age psychiatry, CHU Nice – Université Côte d’Azur, Nice, France

## Abstract

**Abstract:**

The management of dementia presents significant challenges due to the multifaceted nature of major neurocognitive disorders, encompassing cognitive impairments, behavioral and psychological symptoms, and loss of autonomy. Current strategies, including pharmacological and non-pharmacological approaches, have shown limited success, often hindered by adverse effects, lack of efficacy, and insufficient human resources. In this context, emerging technologies offer promising solutions for improving dementia care. Their potential benefits and limitations will be presented: Digital Solutions • Serious Games and Smart Apps: Digital tools can enhance cognitive function and support social engagement for individuals with dementia. Studies show that structured use of these technologies in home settings improves well-being and reduces isolation. Internet of Things (IoT) • Applications: IoT devices such as sensors, GPS trackers, cameras, and wearable technologies are increasingly used to monitor aspects of dementia care. These include activities of daily living (ADLs), sleep patterns, medication adherence, vital signs, and safety concerns like fall detection and wandering.

**Disclosure of Interest:**

None Declared

